# Imaging Characteristics of Diffuse Idiopathic Skeletal Hyperostosis: More Than Just Spinal Bony Bridges

**DOI:** 10.3390/diagnostics13030563

**Published:** 2023-02-03

**Authors:** Iris Eshed

**Affiliations:** Department of Diagnostic Imaging, Sheba Medical Center, Tel Hashomer, Affiliated with the Sackler School of Medicine, Tel Aviv University, Tel-Aviv 5265601, Israel; iriseshed@gmail.com

**Keywords:** DISH, spine, sacroiliac joints, entheses, osteophytes, radiograph, CT, MRI, US

## Abstract

Diffuse idiopathic skeletal hyperostosis (DISH) is a systemic condition characterized by new bone formation and enthesopathies of the axial and peripheral skeleton. The pathogenesis of DISH is not well understood, and it is currently considered a non-inflammatory condition with an underlying metabolic derangement. Currently, DISH diagnosis relies on the Resnick and Niwayama criteria, which encompass end-stage disease with an already ankylotic spine. Imaging characterization of the axial and peripheral skeleton in DISH subjects may potentially help identify earlier diagnostic criteria and provide further data for deciphering the general pathogenesis of DISH and new bone formation. In the current review, we aim to summarize and characterize axial and peripheral imaging findings of the skeleton related to DISH, along with their clinical and pathogenetic relevance.

## 1. Introduction

Diffuse idiopathic skeletal hyperostosis (DISH) is a bone-forming disease characterized by excessive new bone formation in the axial and peripheral skeleton. In the axial skeleton, DISH usually affects the thoracic spine, and in the peripheral skeleton, new bone formation affects entheseal sites, mainly in the pelvis [1,2,3].

DISH is primarily observed in adults older than 45 years, with a male preponderance [4] and an association with metabolic syndrome, obesity, hypertension, and diabetes mellitus [5]. It affects all populations; however, its prevalence is thought to be highest in developed countries. A prevalence directly correlated with the age of 4–32% of DISH was reported among the general population [6].

DISH may be asymptomatic or may manifest as back and cervical pain, dysphagia, pain at peripheral entheseal sites, and limitation of motion of the spine, often mimicking ankylosing spondylitis (AS) [7]. In addition, due to their ankylosed spine, individuals with DISH are susceptible to fractures, even following minor, low-energy trauma [8,9]. Thus, diagnosing DISH in such patients could facilitate early treatment and prevent related complications.

A diagnosis of DISH relies on the radiographic criteria of Resnick and Niwayama, which require flowing osteophytes over at least four contiguous vertebrae of the thoracic spine, preservation of the intervertebral disc space without extensive degenerative intervertebral disease, and the absence of apophyseal and costovertebral joint ankylosis and sacroiliac joints (SIJs) erosion, sclerosis, or bony fusion [1]. These criteria represent a late, end stage of DISH; a decade may pass from the onset of radiographic changes to the formation of characteristic bridging osteophytes [10]. Ustinger suggested a lower threshold of flowing osteophytes over three contiguous vertebrae and added the presence of pelvic enthesophytes to the criteria in order to potentially diagnose DISH earlier [11]. Several additional criteria have been suggested with the aim of diagnosing DISH in earlier stages; however, these criteria have not been widely accepted, with the Resnick and Niwayama criteria remaining the most commonly used criteria [12,13].

The pathogenesis of DISH is not well understood. It is hypothesized that there is, on one hand, excess of growth factors (e.g., insulin, insulin-like growth factor 1, transforming growth factor-β1, and more) inducing the transformation of mesenchymal cells into fibroblasts and osteoblasts, and on the other hand, there is reduced inhibition of bone-promoting peptides (e.g., matrix Gla protein, bone morphogenic protein-2 inhibition, and Dickkopf-1) [14].

DISH is considered a non-inflammatory condition with an underlying metabolic derangement that results in new bone formation [5,15]. However, an inflammatory component has been suggested due to the similar tendency for the new formation of spinal and peripheral entheses observed for DISH and spondyloarthritis (SpA) [16]. Indeed, imaging studies have not only greatly facilitated early diagnosis; they have also enhanced our understanding of SpA pathogenesis [17,18,19,20,21,22].

Therefore, imaging characterization of the axial and peripheral skeleton in DISH subjects may help to develop earlier diagnostic criteria and provide further data for deciphering the pathogenesis of DISH and new bone formation.

The body of knowledge regarding imaging characteristics of DISH has grown over the last decade, leading to a change in perception regarding DISH, and from a merely radiological entity of doubtful significance, it is now commonly accepted that diagnosis of DISH is of important clinical significance.

In the current review, we aim to summarize the most up-to-date reports characterizing axial and peripheral imaging findings of the skeleton related to DISH, along with their clinical and pathogenetic relevance.

## 2. Axial Skeleton

### 2.1. New Bone Formation in the Spine

**Figure 1:**
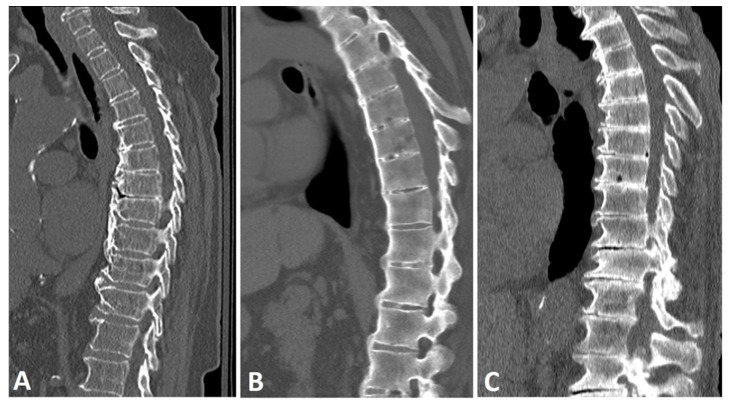
Sagittal CT reconstructions of the thoracic spine in three different patients: (**A**) a patient with DISH with thick, flowing, horizontally oriented, coarse osteophytes; (**B**) a patient with AS with slender, vertically oriented syndesmophytes; and (**C**) a patient with degenerative disc disease with horizontally oriented, non-flowing osteophytes. Note that the disc space is relatively preserved in the DISH subject, but not in the patient with a degenerative spine.

A characteristic feature of DISH is the presence of paraspinal flowing osteophytes resulting from the ossification of soft-tissue structures surrounding the vertebrae [1]. A lucent line extending between the vertebral body cortex and the osteophyte distinguishes these paraspinal osteophytes from degenerative, marginal osteophytes, which are an integral extension of the vertebral endplate, and from SpA-related syndesmophytes, which result from ossification of Sharpey’s fibers of the annulus fibrosis (Figure 1) [23].

The differences in soft-tissue calcification result in two types of osteophytes: vertically oriented syndesmophytes, which are typical of AS, and horizontally oriented osteophytes, which are characteristic of DISH [24]. However, these two types of bone-forming outgrowths have been reported to co-occur in both diseases at different frequencies, i.e., with more horizontal osteophytes in DISH and more vertical syndesmophytes in AS [9,25]. Many studies have reported that the prevalence of DISH increases with age [26,27,28]. However, bone growth has been reported as being significantly more prevalent in subjects younger than 70 years compared with older individuals [29], most likely because the bone growth potential in older patients has already reached its maximum and is approaching the end stage. For DISH, it has been reported that osteophytes bridge over one vertebral space within an average of 10 years [10], a growth rate similar to that reported in AS [21]. Similarities in bone production between DISH and AS have raised the hypothesis that the two diseases may share some features of local or systemic inflammatory pathogenesis [10,16,24]. Indeed, several magnetic resonance imaging (MRI) studies have reported inflammatory bone marrow edema corners and fat metaplasia corners, findings characteristic of SpA, in the spine of subjects with DISH [30,31]. However, the prevalence of such lesions is relatively low and could be attributed to spinal degeneration in general, and not necessarily to DISH per se.

### 2.2. Cervical Spine

**Figure 2:**
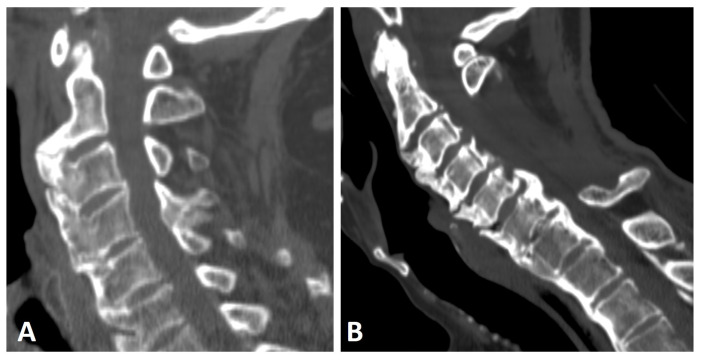
Sagittal CT reconstruction images of two patients with thoracic (not shown) and cervical DISH. (**A**) Flowing chunky osteophytes are located anteriorly to the vertebrae, forming a “candle flame” or “parrot-beak” image. (**B**) Thick, not-yet-flowing anterior osteophytes and thick posterior osteophytes at the C5–6 level.

Anterior ossification of the spine in DISH may involve the cervical spine, primarily along the lower half of the anterior border of the vertebral body, forming a “candle flame” or “parrot-beak” image (Figure 2) [32]. These cervical flowing osteophytes are frequently asymptomatic but may result in dysphagia, sleep apnea, airway obstruction, and difficulty in intubation [7,23,33]. It has been reported that approximately one-third (33%) of subjects with DISH involving the middle or lower thoracic region have coexisting DISH in the cervical spine, according to whole-spine computed tomography (CT) [34].

In DISH subjects, bridging osteophytes in the cervical spine primarily occur anteriorly and are symmetrically distributed relative to the midline of the vertebral bodies [35]. In contrast, in the thoracic spine, osteophytes are typically asymmetrical and anterolaterally located [25,36]. It has been suggested that vascular structures act as a natural barrier for the formation of flowing osteophytes in the thoracic spine in DISH [25,29,36]. Thus, the different patterns of new bone formation between the cervical and thoracic spine may stem from differences in vascular anatomy.

**Figure 3:**
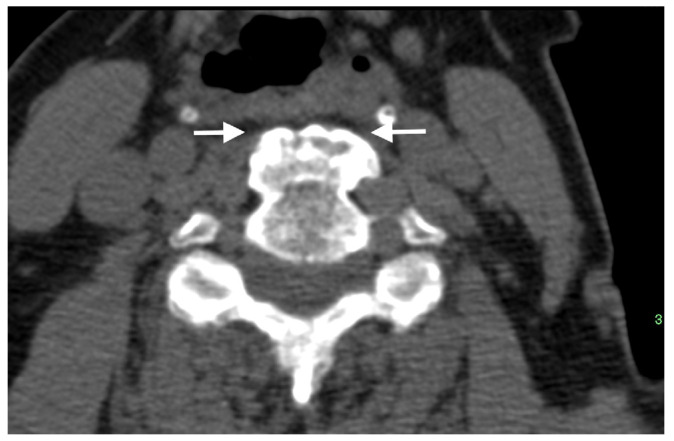
Axial CT image of a thick anterior osteophyte in the cervical spine of a patient with DISH that is impinging and causing narrowing of the adjacent esophagus (arrows).

The chunky anterior cervical osteophytes in DISH are known to impinge upon the anteriorly located airways and esophagus and cause airway obstruction and dysphagia (Figure 3) [33], sometimes requiring surgical intervention [26,37].

**Figure 4:**
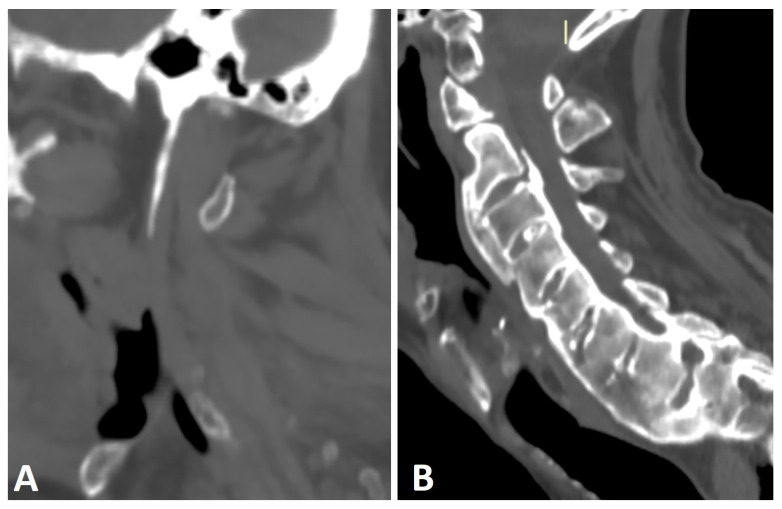
Sagittal CT reconstructions of the cervical spine of two patients with thoracic DISH (not shown). (**A**) The elongated styloid process results from calcification and ossification of the stylohyoid ligament. (**B**) Flowing osteophytes characteristic of DISH accompanied by OPLL.

Another phenomenon known to coexist with DISH is an elongation of the styloid process, which results from entheseal calcification and ossification of the stylohyoid ligament (Figure 4A) [38,39,40,41]. In rare cases, this elongation has been reported to cause craniofacial or cervical pain, termed Eagle syndrome [42]. In a study comparing the length of the styloid process, as measured by CT, between subjects with DISH (as per Resnick and Niwayama criteria), subjects with AS (as per modified New York criteria), and healthy controls, the average lengths of the styloid process in DISH and AS were similar but significantly greater than that of the controls [41]. Moreover, significantly more subjects with AS (30%) and DISH (25%) had an elongated styloid process (>3 cm) than the control group [41]. However, no correlation was seen between the presence of characteristic bone bridging osteophytes in the cervical spine of DISH subjects and an elongated styloid process. Enthesopathy, which is a common feature in both DISH and AS, distinguishes these patients from healthy subjects by causing styloid process elongation. Enthesopathy in AS patients is thought to be of an inflammatory nature [43]. Although enthesopathy in DISH has been classically attributed to mechanical or degenerative causes, it has been suggested that subclinical inflammation is also a cause of cervical enthesopathy in DISH [7,16].

Ossified posterior longitudinal ligament (OPLL) is a hyperostotic condition that may lead to spinal canal stenosis and, potentially, neurological manifestations of varying degrees [44]. OPLL is a distinct entity that may appear without accompanying pathologies, with an incidence of up to 4% [45,46]. It has been reported to be associated with DISH, AS, and diabetes mellitus [45,46], with a concomitance rate of 57.14% for cervical OPLL accompanying DISH (Figure 4B) [47]. Both DISH and OPLL are primarily observed in elderly males and have a reported association with low glucose tolerance and obesity [47]. A shared local inflammatory pathogenesis has been suggested as the basis of both entities; however, this hypothesis needs further corroboration [48].

### 2.3. Thoracic Spine

The thoracic spine is the main segment of the spine affected by DISH and to which the Resnick and Niwayama classifications apply [1,34,49]. One of the exclusions in these criteria is a reduced intervertebral disc height, which indicates degenerative disc disease. However, in their original report, Resnick et al. stated that some degree of intervertebral disc degeneration may be apparent due to the coexistence of both DISH and degenerative disc disease [1]. Indeed, a reduced intervertebral disc height was detected on CT examinations of the thoracic spine of DISH subjects compared with gender- and age-matched controls without DISH [50].

**Figure 5:**
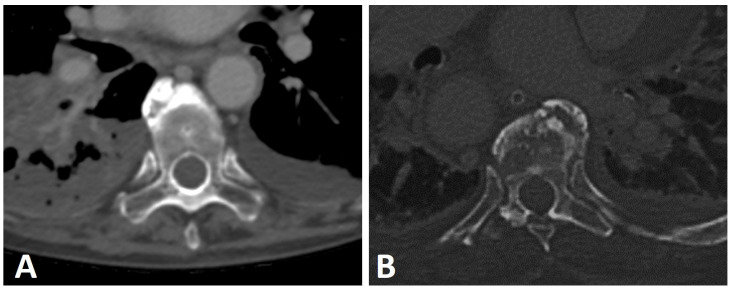
Axial CT images of two patients with thoracic DISH (not shown). (**A**) left-sided and (**B**) right-sided aortas with an anterior osteophyte located contralateral to the aorta’s position.

Bridging osteophytes in DISH are located along the anterolateral aspect of the vertebral bodies, most commonly involving the seventh through eleventh thoracic vertebrae [36,51,52]. These osteophytes are mostly detected on the right side of the spine, leading to the hypothesis that the pulsating, left-sided descending aorta inhibits new bone formation on the left [29,53]. Providing further validation of this hypothesis on the protective effect of the aorta, a previous study demonstrated the same effect in patients with the right-sided aorta, in which the majority of osteophytes were located contralateral to the descending aorta’s location (Figure 5) [25]. Moreover, a study on the location of syndesmophytes in subjects with AS reported the same reduced frequency of syndesmophytes at the vertebral rim near the aorta [54].

In two studies that evaluated CT examinations for thoracic spinal DISH evolution, the amount of newly formed bone increased consistently over time [10,55]. Both studies described the beginning of bony outgrowths at vertebral bodies on either side of the intervertebral space, which, over time, connected to form complete flowing bridges.

**Figure 6:**
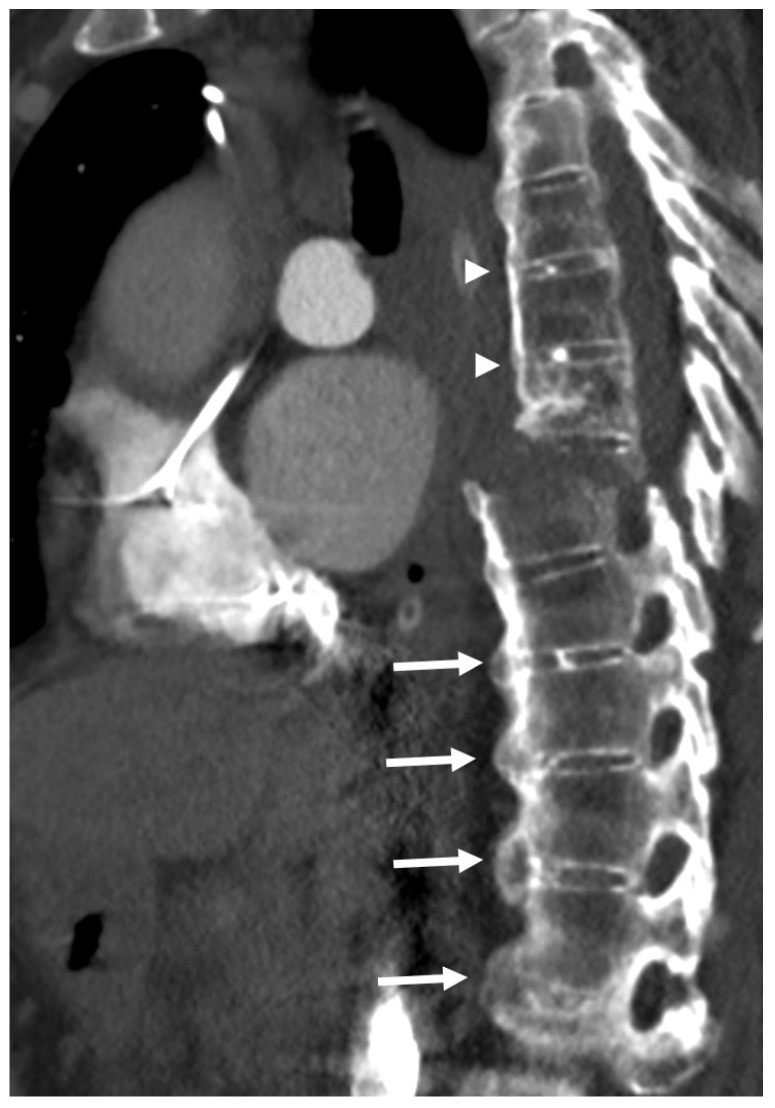
Sagittal CT reconstruction of the thoracic spine of a 78-year-old patient with thoracic DISH after low-energy trauma, showing an unstable extension-type fracture of the mid-thoracic spine. In the given slice, there are two vertically oriented bony bridges in the upper thoracic spine (arrowheads) that may lead to the misconception that this is a patient with ankylosing spondylitis; however, there are clearly many flowing osteophytes compatible with DISH in the lower part of the thoracic spine (arrows).

An ankylosed spine, such as that observed in DISH, is rigid and, as a result, is susceptible to injury, even from low-energy trauma. DISH is associated with a prevalence of thoracolumbar vertebral fractures of 4–18%, of which multilevel fractures are reported in about 8% [56,57]. These fractures are frequently extension-type fractures and are associated with a greater instability risk for spinal cord injury of up to 58% and a higher rate of complications (Figure 6) [58,59,60]. Fractures in DISH pass through the vertebral body, which is the most exposed and weakest point in the ankylosed spine [61]. Detection of vertebral fractures on radiographs of the spine of DISH subjects is challenging; thus, it has been suggested that whole-spine CT be performed in emergency response units in order to prevent negative consequences in DISH subjects, even after minor trauma [9,62].

### 2.4. Lumbar Spine

The lumbar spine is the least affected and most likely the last affected segment of the spine in DISH [34]. When DISH is present in the middle or lower thoracic region, the prevalence of coexisting DISH in the lumbar region is reported at approximately 30%.

There is clinical significance to the involvement of the lumbar spine in DISH. In patients that undergo surgical treatment due to lumbar spinal stenosis, the coexistence of DISH increases the re-decompression rate and expansion of the decompression range [63,64]. In addition, DISH is associated with increased incidence of pseudarthrosis after lumbar spinal fusion [65].

### 2.5. Sacroiliac Joints

**Figure 7:**
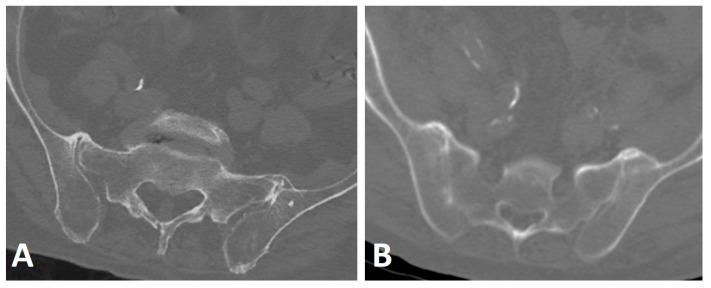
Axial CT images of the SIJs of two patients with DISH. (**A**) Characteristic extra-articular bridging osteophytes are seen anterior to the SIJs. (**B**) In addition to the ventral extra-articular osteophytes, clear ankylosis can be observed within the right and left SIJs.

To distinguish between SpA and DISH, the SIJs appear as an exclusion criterion in the Resnick and Niwayama criteria, so that findings compatible with sacroiliitis, such as erosions, sclerosis, and ankylosis of the SIJ on pelvic radiographs, preclude the diagnosis of DISH [1]. In subsequent studies, Resnick and co-workers described the presence of SIJ osteophytes, para-articular bony bridging, and coexisting osteoarthritis on pelvic radiographs of subjects with DISH [4,66]. Over time and with advances in imaging technology, the characterization of SIJ involvement in DISH has been refined for both CT and MRI. Extra-articular bridging osteophytes located ventrally to the SIJs, similar to flowing osteophytes in the spine, are commonly seen in DISH patients, resulting from enthesopathy of the involved ligament (Figure 7A) [67,68]. This anterior SIJ bridging overlying the SIJ on pelvic radiographs obliterates the joints, resulting in a false diagnosis of SIJ ankylosis, and thus SpA [67,69]. Indeed, studies evaluating the SIJs of subjects with DISH on CT have shown that anterior bridging of the SIJ is a common finding [70,71], but in contrast with the Resnick and Niwayama criteria, both intra-articular and posterior entheseal ankylosis are also prevalent in DISH (Figure 7B) [70,71].

## 3. Pelvis and Appendicular Skeleton

Pelvic and appendicular enthesopathy is common, characteristic extra-spinal manifestation of DISH [72]; consequently, it has been suggested that the spinal involvement threshold be reduced from four to two consecutive vertebrae in the presence of prominent extra-spinal enthesopathy for the classification of DISH [73].

**Figure 8:**
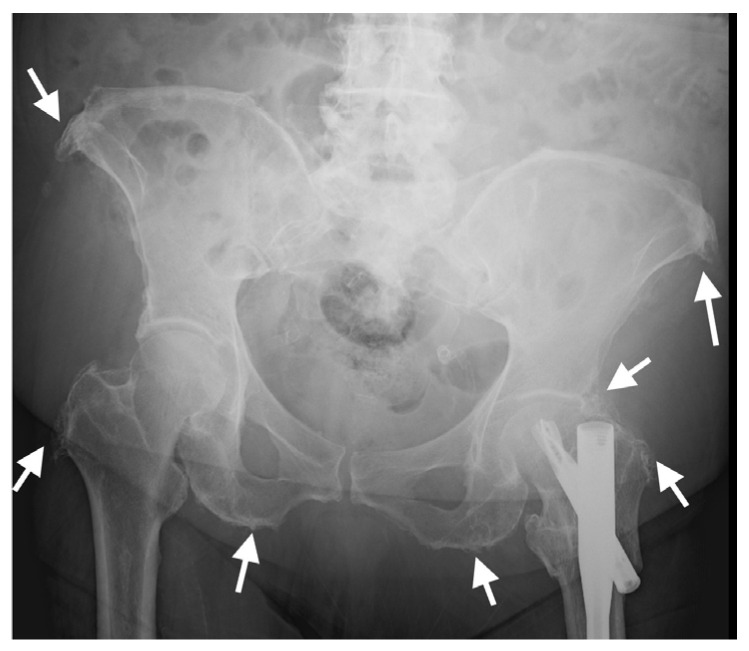
AP radiograph of the pelvis of an 82-year-old female with thoracic DISH (not shown). Characteristic whiskering of the anterior superior iliac spine and ischial tuberosities (arrows) is present on both sides, resulting from enthesopathy in these regions.

Enthesopathy of the sacrotuberous and iliolumbar pelvic ligaments and insertional enthesopathy of tendons such as the iliopsoas, as seen on pelvic AP radiographs (Figure 8), have been shown to be a good indicator of the presence of radiographic spinal DISH [74,75]. A similar association was described for CT examinations of the pelvis, in which enthesopathy was significantly more prominent in subjects with DISH compared with controls for all entheses evaluated (anterior superior iliac spine (ASIS), pubis, ischial tuberosity, greater trochanter), and enthesopathy at the ASIS and greater trochanter significantly distinguished DISH patients from controls [3].

Enthesopathy of the appendicular skeleton is a characteristic feature of DISH and has been found to be more prevalent in DISH subjects versus subjects without DISH [70]. Appendicular skeleton enthesopathy-related lesions have been described using either radiography or ultrasound in the knees, ankles, elbows, and hands of patients with DISH [76,77].

**Figure 9:**
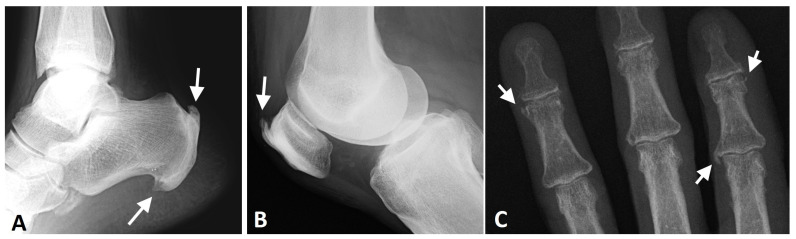
Radiographs of the ankle, knee, and hand of a patient with thoracic DISH (not shown). (**A**) Lateral radiograph of the ankle, showing coarse, thick enthesophytes in the attachment of the Achilles tendon and plantar fascia to the calcaneus (arrows). (**B**) Lateral knee radiograph showing thick, coarse enthesophytes (arrow) in the attachment of the quadriceps tendon to the patella. (**C**) AP radiograph of the fingers, showing enthesophytes in the medial side of the 2nd metacarpophalangeal joint and 4th distal interphalangeal joint and the lateral side of the 2nd distal interphalangeal joint (arrows).

Appendicular DISH is characterized by the involvement of joints that are not commonly affected by primary osteoarthritis (e.g., elbows, shoulders, anterior chest wall, etc.) [32,78] as well as prominent, thick peripheral enthesopathies with calcification and ossification of extra-articular tendinous entheses such as in the tibial tuberosity, olecranon, and Achilles insertion (Figure 9) [79]. In addition, the affected joints in DISH show the exuberant new bone formation and para-articular osteophytes [66]. These changes may result in a reduced range of motion in the affected joints and potentially the subsequent development of degenerative changes [4,75,79].

A clinical radiographic study divided subjects with DISH and/or pelvic enthesopathy into three distinct phenotypes: (1) spinal DISH as per the Resnick and Niwayama criteria and <3 peripheral enthesopathies, (2) extensive spinal DISH as per the Resnick and Niwayama criteria and extensive peripheral enthesopathy, and (3) ≥3 peripheral enthesopathies but no spinal DISH, as per the Resnick and Niwayama criteria [12]. In their report, groups one and two consisted of predominantly elderly males, while group three included mainly females of a younger age. Their results suggest a different phenotype of DISH for women that may be underdiagnosed [13].

## 4. DISH vs. SpA

DISH and SpA are both bone-producing ankylosing diseases involving the axial and appendicular skeletons, in which enthesopathy is a major feature. However, while SpA is considered an inflammation-based disease, DISH is regarded as mechanical or degenerative in nature, although it has been suggested that local inflammation may also play a role in its development [16]. The differentiation between DISH and SpA is usually straightforward due to their distinct clinical features, such as age and genetic predisposition. Nevertheless, clinical similarities between DISH and SpA, such as limited spinal mobility and postural abnormalities, may entail the use of imaging to differentiate between the two conditions [59]. The differences and similarities between DISH and SpA are presented in Table 1.

**Figure 10:**
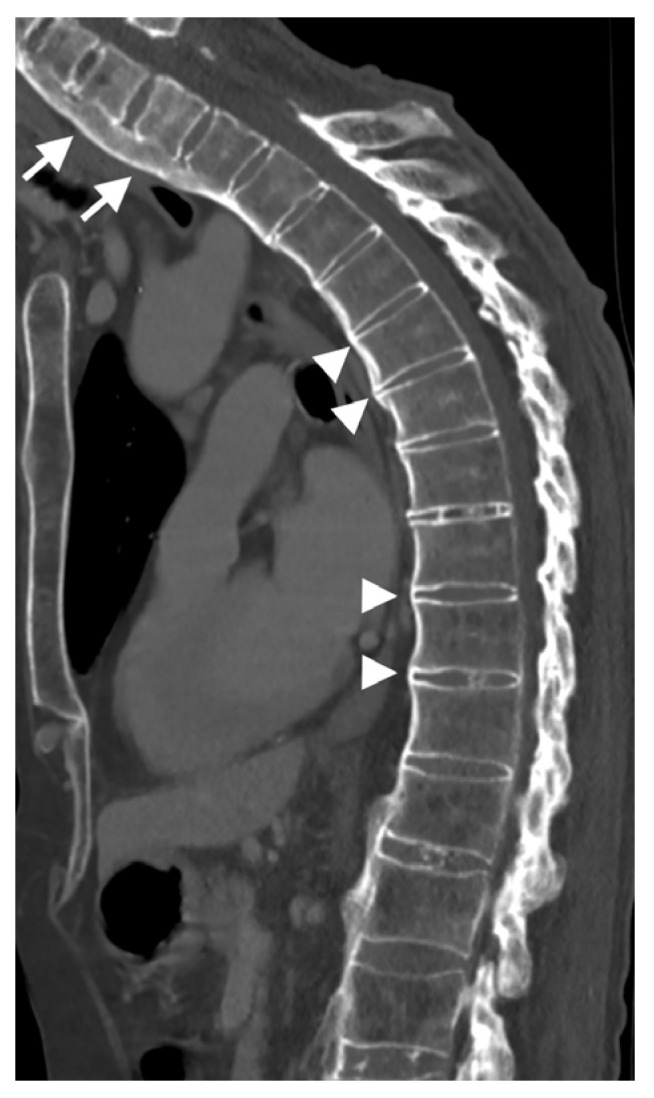
A sagittal CT reconstruction of the cervical and thoracic spine of a 60-year-old patient. There are thick-flowing osteophytes on the cervical spine (arrows), while thin slender vertical syndesmophytes (arrowheads) are in the thoracic spine.

Generally speaking, new bone formation and enthesophytes in the spine and the appendicular skeleton are thick and prominent in DISH but thin in SpA. In addition, DISH-related osteophytes in the spine are primarily located on the right and are horizontal in nature, while in SpA, they are vertically oriented with no predilection to any side [24,66]. However, in many cases, differentiating between DISH and SpA via imaging is not as straightforward as expected. The coexistence of both DISH and AS has been described in several case studies (Figure 10) [80].

This coexistence may result from the fact that both bridging osteophytes and syndesmophytes have been reported to appear in both diseases, although with different ratios, e.g., a majority of syndesmophytes in AS and a majority of right-sided bridging osteophytes in DISH [24]. Another source of confusion is the presence of parasyndesmophytes, which are thick para-spinal osteophytes seen in patients with psoriatic arthritis (PsA) [81,82]. DISH has been reported to appear in 8% of patients with PsA, comparable to the prevalence reported in the general elderly population [83]. Syndesmophytes in PsA are asymmetrical and para-marginal, resulting from inflammation and remodeling processes at the disco-vertebral junction, leading to gradual ossification of the periphery of the annulus fibrosus and the formation of vertical bony bridges [84]. In contrast, bridging osteophytes result from ossification along the anterior longitudinal ligament, the paravertebral connective tissue, and the periphery of the annulus fibrosus [85]. Thus, parasyndesmophytes are usually easy to detect and differ from the bridging osteophytes of DISH in appearance and location. However, the co-occurrence of both entities is not uncommon in the relevant age demographic.

Partial or complete ankylosis of the apophyseal joints has been described in advanced AS [86], whereas in a previous study on DISH, the main pathology described in these joints was degenerative joint disease similar in appearance and prevalence to patients without DISH [50]. Yet, in the same study, entheseal bone formation identical to that observed in AS was described in the costovertebral joints of patients with DISH [50]. Thus, further research characterizing and comparing the involvement of the apophyseal and costovertebral joints in DISH and AS is warranted.

Many studies describing the co-occurrence of DISH and SpA have based their diagnosis of SpA on abnormalities seen at the SIJ, whereas their presence is an exclusion criterion in the Resnick and Niwayama classification for DISH [66,80]. However, we and others have shown that, similar to SpA, the SIJ of subjects with DISH is commonly abnormal on CT, with enthesophytes and ankylosis in ligament attachments both outside and within the joints [70,71]. Thus, diagnosing SpA based on the appearance of these abnormalities in the SIJ is erroneous. Hence, it is not yet fully clear whether there is a real overlap between DISH and SpA or whether this is merely a result of misconceptions regarding the inclusion or exclusion imaging criteria for DISH.

One clear difference between DISH and SpA is the presence of SIJ erosions. These are common features of SpA but are seldom seen in DISH [70]. Therefore, the presence of sacroiliac erosions can clearly exclude a diagnosis of DISH, but either partial or total ankylosis of the SIJ cannot.

Pelvic enthesopathy on radiographs in DISH has a distinct appearance, with hypertrophic whiskering of entheseal attachments, whereas in SpA, bone proliferation is milder and is associated with bony erosion and sclerosis [87]. Moreover, while prominent enthesophytes and bone production are seen around the hip joints in DISH, the principal radiographic findings in SpA are concentric joint space narrowing, erosions, and ankylosis, which are uncommon in DISH [87].

Several studies have described the MRI appearance of the spine and SIJs in DISH. Here, bone marrow edema and fat in the vertebral corners comparable to those characteristic of SpA have been described. However, their prevalence is much lower compared with SpA, and has been attributed to a degenerative process rather than an inflammatory process [30,31,88]. Bone marrow edema in the SIJ has also been observed in subjects with DISH; however, these observations are uncommon, to a lesser extent than that seen in SpA. Again, this is most likely due to a degenerative joint process rather than a similar inflammatory pathogenesis [31].

## 5. Conclusions

DISH is not solely an imaging entity, but can manifest with significant clinical consequences, including spinal fractures even from low-energy trauma, neurologic deficits resulting from spinal stenosis, and dysphagia or airway obstruction due to abutting anterior cervical bridging osteophytes. In addition, diagnosing DISH and differentiating it from SpA is important for facilitating correct and targeted treatment. It is therefore important for clinicians to recognize the imaging characteristics of DISH in the axial and peripheral skeleton and understand DISH’s clinical consequences, evaluation, and management.

The body of knowledge regarding imaging characteristics has grown over the last decade. The current Resnick and Niwayama criteria for DISH correspond to an end-stage diagnosis of the disease, in which the spine is already ankylosed. A newer set of classification criteria is warranted for diagnosis in an earlier, pre-ankylotic stage of the disease.

The pathogenesis underlying this disease is still unclear, and although it is thought to be a degenerative disease, it has been suggested that similarities to SpA may imply an inflammatory basis. Imaging studies further characterizing the disease may potentially aid in deciphering the currently obscure pathogenesis of DISH.

## Figures and Tables

**Table 1:** DISH vs. Spondyloarthritis: differences and similarities.

	**DISH**	**SpA**
**Age**	Elderly (>50 years)	Young adults (>20–40)
**Gender**	M > F	M > F
**Pathogenesis**	Mechanical/localized inflammation	Inflammatory
**Enthesopathy**	Axial and peripheral	Axial and peripheral
	Thick and pronounced With no accompanied erosions and sclerosis	Thin, with accompanied erosions and sclerosis
**Spine**		
Bone production	Thick, bridging horizontal osteophytes	Thin, slender, vertical syndesmophytes
**MRI**		
BME corners	Present but uncommon	Common
Fat corners	Present, not rare	Common
Periarticular fat metaplasia	Rare	Common
**Sacroiliac joints**		
Bone production	Bridging osteophytes Anterior to SIJ	Intra-articular bridging osteophytes
Erosions	Rare	Common
**MRI**		
Periarticular BME, suggestive of SpA	Rare Upper part of SIJ	Common Along entire SIJ
Periarticular fat metaplasia	Rare	Common

DISH, diffuse idiopathic skeletal hyperostosis; SpA, spondyloarthritis; M, male; F, female; MRI, magnetic resonance imaging; BME, bone marrow edema; SIJ, sacroiliac joint.

## Data Availability

Not applicable.
